# Regional disparities in warm season rainfall changes over arid eastern–central Asia

**DOI:** 10.1038/s41598-018-31246-3

**Published:** 2018-08-29

**Authors:** Wenhao Dong, Yanluan Lin, Jonathon S. Wright, Yuanyu Xie, Yi Ming, Han Zhang, Rensheng Chen, Yaning Chen, Fanghua Xu, Namei Lin, Chaoqing Yu, Bin Zhang, Shuang Jin, Kun Yang, Zhongqin Li, Jianping Guo, Lei Wang, Guanghui Lin

**Affiliations:** 1 Ministry of Education Key Laboratory for Earth System Modeling, Department of Earth System Science, and Joint Center for Global Change Studies (JCGCS), Tsinghua University, Beijing, 100084 China; 2 Geophysical Fluid Dynamics Laboratory, Princeton/NOAA, Princeton, New Jersey 08540-6649 USA; 3 Qilian Alpine Ecology and Hydrology Research Station, Key Laboratory of Inland River Ecohydrology, Northwest Institute of Eco-Environment and Resources, Chinese Academy of Sciences, Lanzhou, 730000 China; 4 State Key Laboratory of Desert and Oasis Ecology, Xinjiang Institute of Ecology and Geography, Chinese Academy of Sciences, Urumqi, 830011 China; 5 State Key Laboratory of Cryospheric Sciences/Tian Shan Glaciological Station, Cold and Arid Regions Environmental and Engineering Research Institute, Chinese Academy of Sciences, Lanzhou, 730000 China; 6 State Key Laboratory of Severe Weather and Key Laboratory of Atmospheric Chemistry of CMA, Chinese Academy of Meteorological Sciences, Beijing, 100081 China; 7 Key Laboratory of Tibetan Environment Changes and Land Surface Processes, Institute of Tibetan Plateau Research, Chinese Academy of Sciences (CAS), and the CAS Center for Excellence in Tibetan Plateau Earth Sciences, Beijing, 100101 China

## Abstract

Multiple studies have reported a shift in the trend of warm season rainfall over arid eastern–central Asia (AECA) around the turn of the new century, from increasing over the second half of the twentieth century to decreasing during the early years of the twenty-first. Here, a closer look based on multiple precipitation datasets reveals important regional disparities in these changes. Warm-season rainfall increased over both basin areas and mountain ranges during 1961–1998 due to enhanced moisture flux convergence associated with changes in the large-scale circulation and increases in atmospheric moisture content. Despite a significant decrease in warm-season precipitation over the high mountain ranges after the year 1998, warm season rainfall has remained large over low-lying basin areas. This discrepancy, which is also reflected in changes in river flow, soil moisture, and vegetation, primarily results from disparate responses to enhanced warming in the mountain and basin areas of AECA. In addition to changes in the prevailing circulation and moisture transport patterns, the decrease in precipitation over the mountains has occurred mainly because increases in local water vapor saturation capacity (which scales with temperature) have outpaced the available moisture supply, reducing relative humidity and suppressing precipitation. By contrast, rainfall over basin areas has been maintained by accelerated moisture recycling driven by rapid glacier retreat, snow melt, and irrigation expansion. This trend is unsustainable and is likely to reverse as these cryospheric buffers disappear, with potentially catastrophic implications for local agriculture and ecology.

## Introduction

Arid Eastern-Central Asia (AECA) is situated in the continental interior, far from the ocean and surrounded by rugged mountain ranges. This geographical setting restricts moisture transport into AECA, resulting in a typical dry continental climate. Water is critical to the well-being of both the arid eco-environment and the people who reside there^1,2^. The uneven distribution of water resources in AECA has contributed to some of the most prominent water allocation problems in the world^3–5^. Competition and conflicts over water use have already arisen on both national and local scales^4,6^, even as rapid population growth compounds existing water stresses^7^. The implementation of the proposed “*Silk Road Economic Belt*” strategy will pose severe challenges to the sustainable management of water resources within AECA, which may in turn diminish the socioeconomic benefits of this project to the countries and regions involved^3,8^. It is therefore necessary to catalogue and understand the processes that affect water supplies in AECA.

Warm season (May–September) rainfall is an important contributor to water resources in AECA. Precipitation during these five months accounts for 50~70% of the annual total. This precipitation, together with meltwater from glaciers and snow, plays a vital role in maintaining the fragile ecosystem^1^ and supporting local agriculture. Although AECA is among the world’s driest regions, both direct observations and reconstructions of precipitation show that warm season rainfall increased significantly in this region over the second half of the twentieth century^9–13^. This increase in rainfall has been projected to continue under global warming^14^, potentially ushering in a new, wetter regional climate with enhanced water availability and greater agricultural productivity. Multiple studies have examined the mechanisms behind the increase of rainfall^9,10,15^. The positive trend has generally been interpreted as a superposition of large-scale circulation changes and long-term atmospheric moistening under global warming^12–14,16^. However, recent studies have warned of an emerging downward trend in warm season rainfall starting around the turn of the new century^6^, potentially linked to changes in the prevailing circulation and moisture transport patterns^12^.

Although area-mean warm season precipitation over AECA has decreased, important sub-regional contrasts in rainfall trends have been identified. Mean annual precipitation has continued to increase over the outer northern and eastern mountain ranges, but has decreased over the inner mountains, particularly during the warm season^1^. Meanwhile, observations indicate an increase in hydrological extremes within the low-lying Tarim Basin^11^ and a dramatic drop in glacier mass within the Tien Shan mountain ranges^1,17^ Given the volatile history and sensitive nature of water resource management in Central Asia, even small changes in water allocation could have large impacts on regional stability and the ecological and economic environments. However, relatively little attention has been paid to regional disparities in rainfall changes between the mountain and basin areas of AECA, and the mechanisms behind these disparities remain unclear. Here, we investigate variations in warm season rainfall over AECA during 1961–2015. We focus on moisture fluxes within the atmosphere, including their convergence and their interactions with the local environmental factors. We then examine different climatic factors in tandem to explore the disparity between the warm season rainfall trend over the Tien Shan mountain ranges and adjacent basin areas.

### Characteristics and changes of warm season rainfall

**Figure 1:**
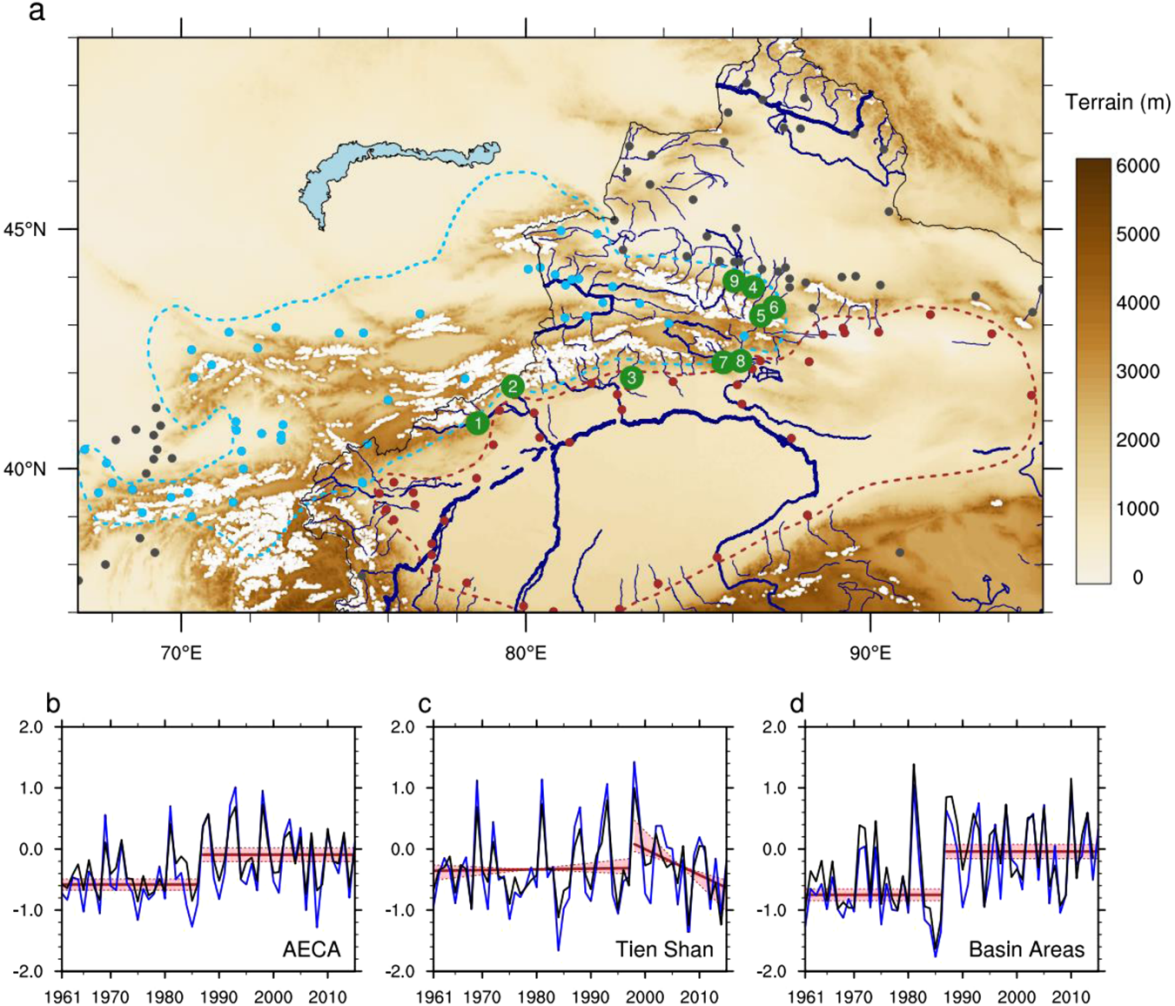
(**a**) Topographic features and hydrological networks in arid eastern–central Asia (AECA). Major rivers in the Chinese portion of AECA are drawn in blue. Glaciers are drawn in white. Solid dots denote weather stations, with cyan stations located in the Tien Shan, brown stations located in basin areas, and grey stations located in other parts of AECA. River runoff measurement stations are indicated with numbers corresponding to the key in Fig. 4. The subregions of AECA discussed in the text are delineated by the cyan dashed line (Tien Shan) and the brown dashed line (basin areas). Time series of standardized warm season precipitation anomalies based on station records (black) and an ensemble of six gridded precipitation datasets (blue) in (**b**) AECA (all dots in **a**), (**c**) the Tien Shan mountains (cyan dots in **a**), and (**d**) basin areas (brown dots in **a**). All data are normalized relative to the mean and standard deviation of the overlapping period (1998–2007) prior to construction of the ensemble. Climate shifts in the time series are marked in red, including changes in the mean state over all AECA and the basin areas in 1987 and a change in the trend over the Tien Shan mountains in 1998 (see Methods for details). Shading and dotted lines in (**b**–**d**) indicate 95% confidence intervals.

The topography of AECA includes high mountain ranges, desert, and loess plateau (Fig. 1a). Inhibition of moisture flow by the Tien Shan imposes a distinct northwest-to-southeast gradient in precipitation rates^1^, with the southeastern part of AECA receiving 30~80 mm of rainfall on average during May–September and the Tien Shan mountain ranges (including the relatively moist northwestern sectors) receiving 100~200 mm (Supplementary Fig. S1a). This spatial gradient is also evident in year-to-year fluctuations of warm season precipitation, with the most pronounced variability located over the Tien Shan (Supplementary Fig. S1b).

Direct observations of precipitation are limited in AECA. Most meteorological stations are situated in the more densely populated periphery of the mountain ranges. Moreover, many stations in the northwestern portion of the AECA region stopped reporting data following the collapse of the Soviet Union. We have compiled data from 147 stations that have provided continuous observations since 1961 (Fig. 1a). Although this represents the best available subset of station-based data, uncertainties due to the inhomogeneous distribution of these stations remain (see discussion below). Warm season precipitation averaged over these 147 stations has generally increased over the past several decades (Fig. 1b), but temporal variations have not been monotonic. Previous studies have identified a shift from less warm season precipitation to more warm season precipitation in 1987^9–11^. Application of a climate change-point detection algorithm^18^ confirms this shift point, with the mean state changing from 82.0 ± 4.9 mm (±s.e.m.) during 1961–1986 to 95.90 ± 6.1 mm during 1987–2015 (*p* < 0.05; Fig. 1b; see Methods). We apply the same change-point detection algorithm to warm season precipitation in the Tien Shan and basin areas separately. This analysis identifies no statistically significant shifts in the mean state over the Tien Shan mountain ranges during 1961–2015 (Fig. 1c), but does reveal a particularly pronounced shift toward larger rainfall over basin areas in 1987^11,16^ (78.1 ± 10.0 to 100.6 ± 8.4 mm; *p* < 0.05; Fig. 1d).

**Figure 2:**
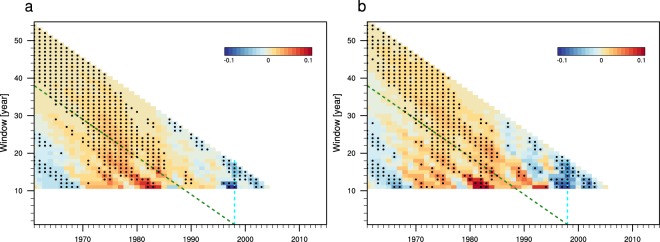
Running window linear trends in normalized warm season rainfall using the robust Theil–Sen estimator during 1961–2015 over the Tien Shan mountains (as in Fig. 1c) using (**a**) station records and (**b**) an ensemble of six gridded datasets. The *x*-axis denotes the start year of the analysis period, while the *y*-axis denotes the length of the analysis period. The dashed green line marks all linear trends that end with the year 1998, while the dashed cyan line indicates trends that start with the year 1998. Black dots indicate trends that are statistically significant at the 95% confidence level. Running windows with lengths less than 10 years are not considered.

**Figure 3:**
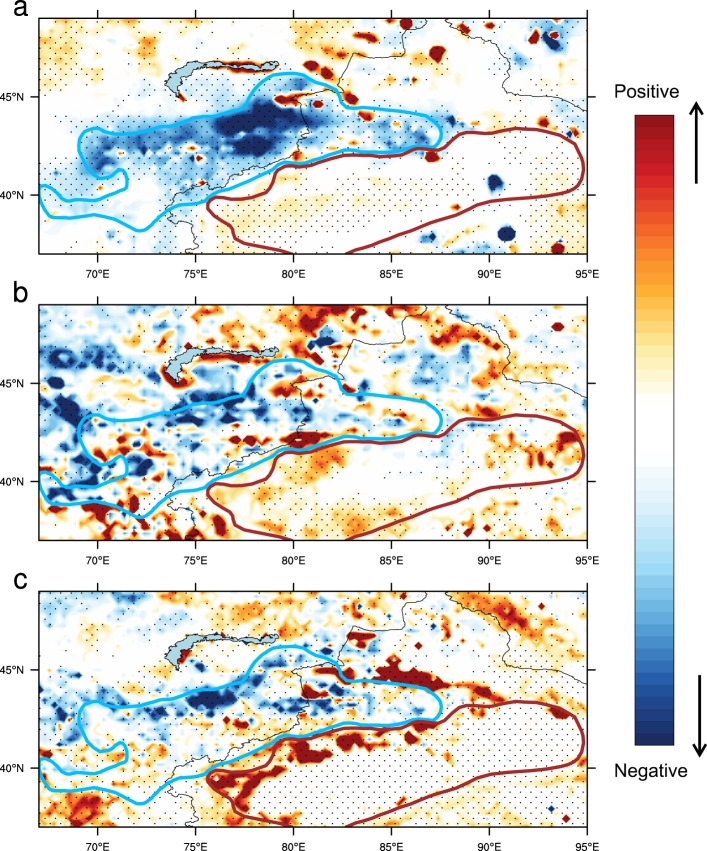
Distributions of linear trends in (**a**) precipitation based on TMPA data set, (**b**) soil moisture and (**c**) NDVI during the summers of 1998–2015. Note that NDVI is only available for the summers of 2000–2015. Stippling indicates that trends are significant at the 95% confidence level. All trends are calculated using the robust Theil–Sen estimator (see Methods). The dashed cyan contour bounds the Tien Shan mountain ranges and the dashed brown contour bounds the basin areas.

Although no significant shift in the mean state is detected over the Tien Shan, precipitation trends over this region change sign around the year 1998 (Fig. 1c). To corroborate the identified shift in rainfall trends, we perform running window trend analyses using the robust Theil–Sen estimator (see Methods) with different starting and ending years (Fig. 2). We restrict our analysis to time windows longer than 10 years to eliminate small-scale fluctuations associated with interannual variability. The results indicate positive trends in warm season rainfall before 1998 regardless of the start year, which then shift to significant negative trends after 1998 regardless of the end year. Application of the change-point detection algorithm (see Methods) confirms that this shift in Tien Shan rainfall trends around 1998 is statistically robust. Results are similar for start years between 1996 and 2003 (Fig. 2), and are therefore not an artefact of the anomalously large precipitation in 1998. Moreover, the shift to a strong decreasing trend after 1998 is evident not only in station data, but also in six different gridded precipitation analyses (Supplementary Table S1). The spatial distribution of post-1998 warm season rainfall trends (Figs 3a and S2) likewise shows strong decreases over most of the Tien Shan mountain ranges, in contrast to slight increases over basin areas. Similar patterns appear in the spatial distributions of trends in surface soil moisture (Fig. 3b) and normalized difference vegetation index (NDVI; Fig. 3c). Trends toward wetter soils and greener vegetation are evident in the basin areas, especially along the periphery of the mountain ranges, while the prevailing trends within the Tien Shan are toward drier soils and lower NDVI. The emergence of fundamentally different trends over the mountain and basin areas after 1998 implies that the mechanisms controlling rainfall variability over these two regions may differ as well, and calls for a careful investigation of the factors that contribute to these discrepancies.

### Recent declines in glacier area and river discharge

**Figure 4:**
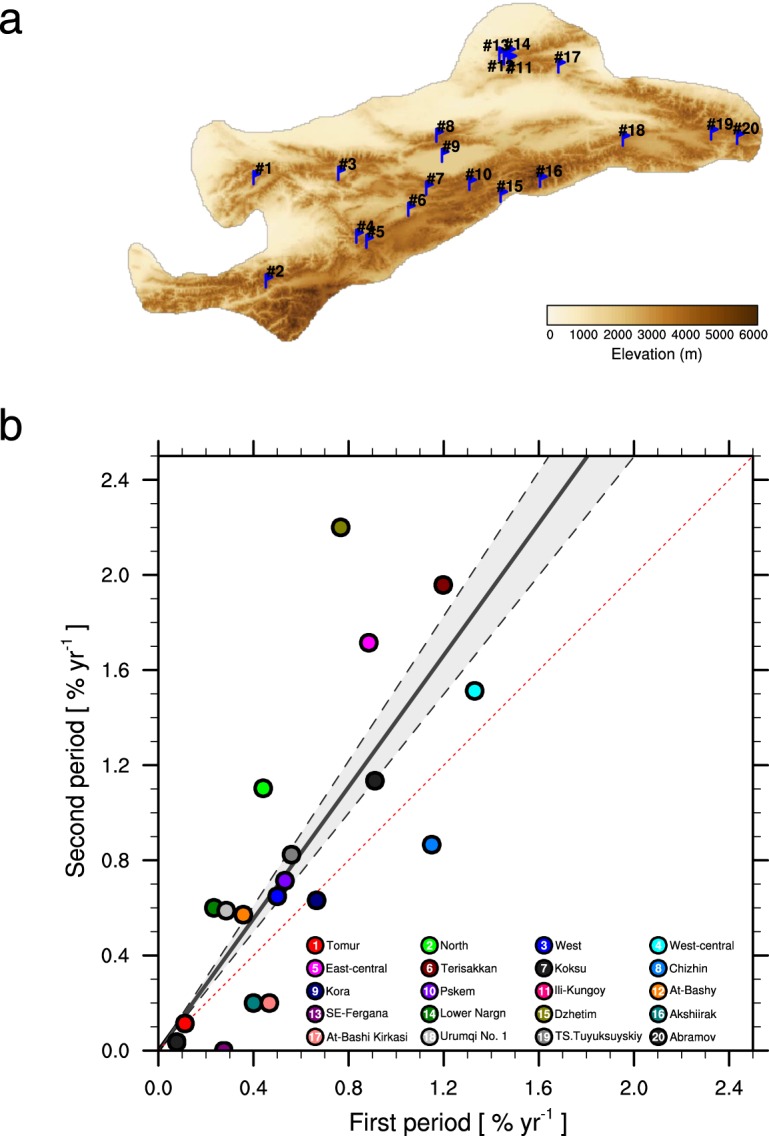
(**a**) Locations of glacier measurement stations over Tien Shan, (**b**) Scatter plot of glacier melting rate calculated during the first period (*x*-axis) and the second period (*y*-axis). The black solid line indicates the least-square linear fit among the glaciers, with the regression equations: *y* = 1.39*x* (*r*^2^ = 0.57, *P* < 0.001). Gray shading and dash lines indicate the stand error of the linear fit. The melting rate are calculated relative to the first measurement year, which assume to be 100% of glacier as the reference year (see Methods for details).

Decreases in warm season rainfall over Tien Shan after 1998 are also evident in observations of river discharge. The response of river flow in catchments with glaciated headwaters to changes in cryospheric climate is complex. Rapid warming in the Tien Shan mountain ranges has accelerated glacier and snow melt in recent years^2,17^. Meltwater from these cryospheric sources is an essential source of water resources downstream^1,2^, with human populations in AECA dependent on glacial contributions to streamflow for irrigation, industry, and hydropower^5,10^. Figure 4 shows the melting rates of 20 glaciers over Tien Shan during two different periods divided around the turn of this century (See Methods). All 20 glaciers show enhanced melting rates since the reference year, with average rates of 0.58 ± 0.35% per year during the first period increased to 0.81 ± 0.61% per year during the past ~15 years. This marks a considerable acceleration (~1.5 times) in the rate of glacier mass loss in the Tien Shan in recent years (*r*^2^ = 0.57, *P* < 0.001), and is consistent with previous studies^2,17^. The recent intensification of glacial melting has been attributed to both natural and anthropogenic causes, including enhanced warming, negative precipitation anomalies^2^, and increased deposition of dust and organic aerosols in snow-covered regions^19^.

**Figure 5:**
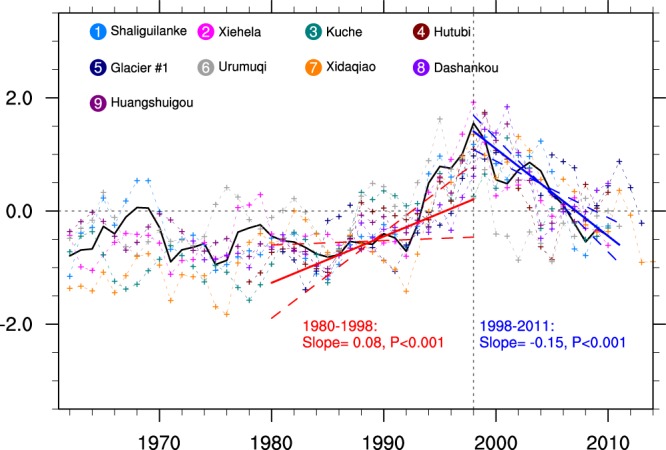
Time series of normalized river runoff originating from glaciated headwaters in the Tien Shan mountain ranges during 1961–2014. Colored marker lines indicate the nine rivers shown in Fig. 1a. All data are normalized relative to the mean and standard deviation of the overlapping period (1980–2011). Trends for each river are calculated using the robust Theil–Sen estimator (see Methods). The black line is the average of two remote montane rivers: *Shaliguilanke* and *Xiehela*. Shading and dotted lines indicate 95% confidence intervals on trends in this time series. Detailed information for these nine rivers is listed in Supplementary Table S2.

Runoff anomalies from nine rivers located in the peripheral regions of the Tien Shan mountains show weak fluctuations before the early 1990s, followed by a sharp increase through the year 1998 (Fig. 5). This increase corresponds to reductions in glacier mass and snow cover due to rapid warming over the Tien Shan^17,20^. In the absence of other factors, accelerated melting should translate into larger river discharge; however, river runoff has decreased significantly over the past ~15 years (Fig. 5 and Supplementary Table S2). Anthropogenic influences, such as expanded water use for irrigation or hydraulic projects, can complicate attributions of changes in river discharge. Decreases in downstream runoff despite increased headwater runoff have been observed in arid regions in situations with increased water use^1^. To minimize the anthropogenic influences, we examine the streamflow in two remote montane rivers located far from human population centers (Fig. 5; black curve). Sharp decreases in streamflow after 1998 are also evident for these two isolated rivers, consistent with the idea that decreased river runoff after 1998 results primarily from the reduction in precipitation over the Tien Shan mountain ranges. It also indicates that these river systems may be reacting more directly to interannual variations in precipitation as their glacial buffers disappear. A better understanding of precipitation variations in AECA, especially over the Tien Shan, is urgently needed to support water resource management in this region.

### Large-scale moisture transport

Large-scale moisture transport is crucial for rainfall over inland arid and semi-arid regions. Distance from the ocean and the physical barriers presented by the surrounding mountains reduce the efficiency of moisture transport from the oceans into AECA, resulting in a dry continental climate. In particular, the high elevation of the Tibetan Plateau severely restricts the transport of moist air associated with the Indian Summer Monsoon into AECA. Transport from the western and northern boundaries is thus the main source of moisture for precipitation over AECA. Supplementary Fig. S3 shows moisture transport at four levels (850 hPa, 700 hPa, 600 hPa, and 500 hPa) that encompass the large variations in surface elevation across AECA. Moisture transport into the basin areas dominates at 850 hPa (Supplementary Fig. S3a), and can be traced back primarily to eastward flow from the North Atlantic Ocean and southward flow from the Arctic Ocean^12,17^. These moist air masses pass between the Altai and Tien Shan mountain ranges and then curl into the Junggar and Tarim Basins. Moisture transport at 700 hPa (Supplementary Fig. S3b) is largest over the outer northern Tien Shan Mountains, but does not supply much moisture to the higher central Tien Shan. Substantial moisture transport into AECA on this level is confined mainly to the region between the Tien Shan and Altai mountain ranges, and to a lesser extent to the Tarim basin. Moisture transport at 600 hPa (Supplementary Fig. S3c) is primarily associated with strong westerlies. The largest moisture transport at this level is to the Tien Shan mountain ranges, with relatively little transport to the basin areas. Moisture transport at 500 hPa (Supplementary Fig. S3d) is primarily located to the south of AECA. We therefore focus on the relationships between warm season rainfall variations and moisture transport in the lower troposphere (850 hPa, 700 hPa, and 600 hPa). This picture of moisture transport is consistent with previous studies^6,12^, in that the primary sources of moisture for warm season rainfall over AECA are eastward and southward advection of moist air from the North Atlantic and Arctic Oceans, supplemented by contributions from inland seas (such as the Black Sea, the Caspian Sea, and the Aral Sea).

We hypothesize that warm season rainfall over the basin areas is modulated primarily by moisture transport at lower levels (850 hPa and 700 hPa), while warm season rainfall over the Tien Shan mountain ranges is modulated primarily by moisture transport in the lower middle troposphere (600 hPa). To test this hypothesis, we examine the co-evolution of changes in moisture transport and changes in warm season rainfall. Based on the climate change point analysis discussed above, the analysis period is partitioned into three parts: 1961–1987, 1988–1998, and 1999–2015. Mean warm season rainfall during 1988–1998 is systematically larger than that during 1961–1987 over both the Tien Shan mountain ranges and basin areas (Supplementary Table S1). This increase in warm season rainfall is consistent with changes in the mean moisture transport on the 850, 700, and 600 hPa isobaric surfaces (Supplementary Fig. S4). Anomalous stronger westerlies favor larger external moisture transport into AECA during 1988–1998 relative to 1961–1987 (the second column in Supplementary Fig. S4). By contrast, moisture transport throughout the lower troposphere was substantially reduced over AECA during 1999–2015 relative to 1988–1998 (the third column in Supplementary Fig. S4), with anomalous easterlies prohibiting the westward moisture transport. These results are consistent with previous conclusions based on regional dynamic recycling simulations^12^. The reduction in moisture transport can explain the warm season rainfall variation in Tien Shan mountain ranges. However, warm season rainfall over basin areas has held approximately steady over the past 15 years despite reduced moisture transport (Figs 1d and 3a, Supplementary Table S1, and Supplementary Fig. S2). Changes in precipitation reflect the combined influences of changes in the large-scale circulation and changes in local moisture recycling. In isolation, a similar large-scale circulation background and associated moisture transport should have resulted in a decrease in precipitation over the basin areas. That no such decrease occurred implies that local moisture recycling intensified to compensate. We discuss this possibility further below.

**Figure 6:**
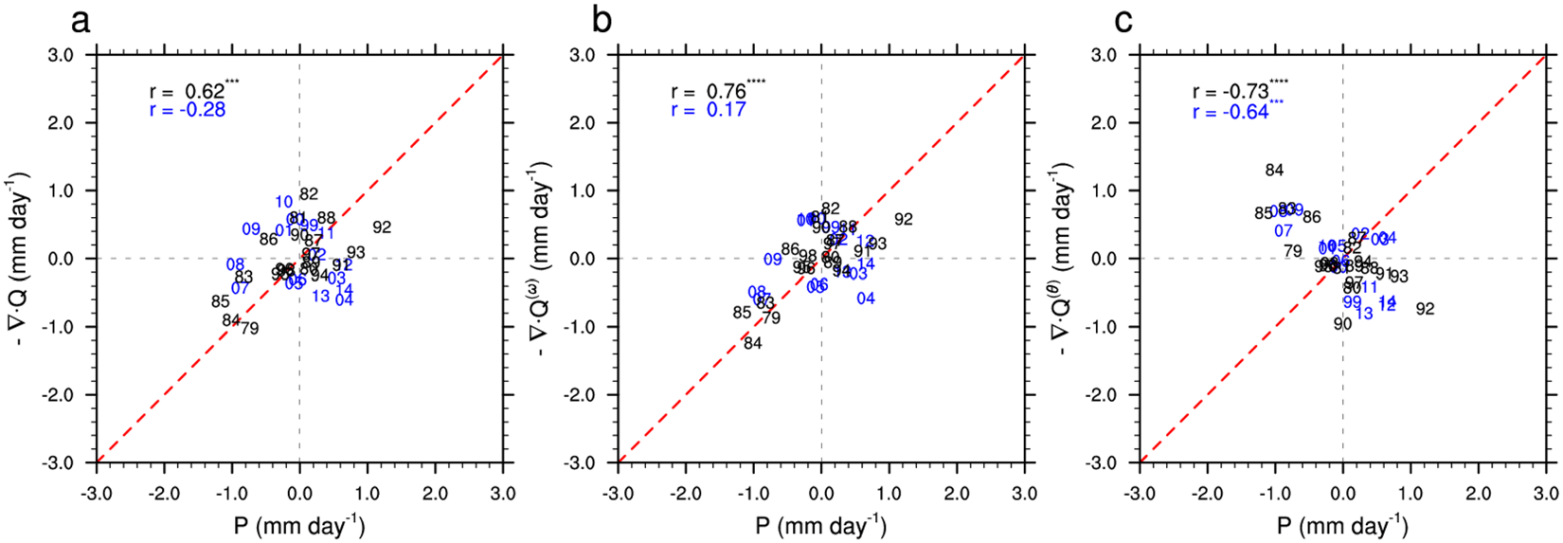
Scatter plots of warm season precipitation anomalies against (**a**) moisture flux convergence (−∇Q) anomalies and its (**b**) dynamic component (−∇Q^ω^), and (**c**) thermodynamic component (−∇Q^θ^) during 1979–2014. Black numbers indicate years before 1998 (using APHRODITE precipitation data), while blue numbers indicate years after 1998 (using TMPA precipitation data). Correlations with three (four) stars are significant at the 99% (99.9%) confidence level.

Moisture fluxes are useful for identifying moisture sources and transport pathways, but precipitation occurs where moisture fluxes converge. Changes in moisture flux convergence depend on both changes in wind fields and changes in moisture content. To explore the mechanisms behind historical variations in warm season precipitation over AECA, we decompose the total moisture flux convergence (−∇Q) into two mutually exclusive components that represent the effects of changes in atmospheric moisture content (−∇Q^θ^) and changes in the large-scale circulation (−∇Q^ω^), respectively (See Methods). Year-to-year variations in moisture flux convergence are tightly correlated with variations in precipitation over AECA during 1979–1998 (*r* = 0.62, *p* < 0.01; Fig. 6a). This relationship is consistent with changes in the large-scale circulation component (*r* = 0.76, *p* < 0.001; Fig. 6b), but opposes changes in the moisture content component (*r* = −0.73, *p* < 0.001; Fig. 6c). Variations in warm season precipitation are therefore primarily controlled by changes in the large-scale atmospheric circulation during this period: without external moisture transport, local moistening of the atmosphere could not have sustained the observed increase in precipitation. More generally, increases in moisture flux convergence due to changes in the large-scale circulation favor increased precipitation, while increases in moisture flux convergence due to increased atmospheric moisture content are associated with decreased precipitation, as discussed below. The correlations for 1979–1998 are consistent with previous studies, which have emphasized that circulation changes are the dominant control on interannual variability in summer precipitation over AECA^14^. It is therefore surprising that correlations between moisture flux convergence and warm season precipitation are insignificant after 1998, apart from the moisture content component (*r* = −0.64, *p* < 0.01; Fig. 6c). Despite the anti-correlation, the strength of the relationship implies that variations in warm season precipitation after 1998 are tightly connected to thermodynamic changes, even as they appear to be largely decoupled from changes in the large-scale circulation.

### Local environmental factors

**Figure 7:**
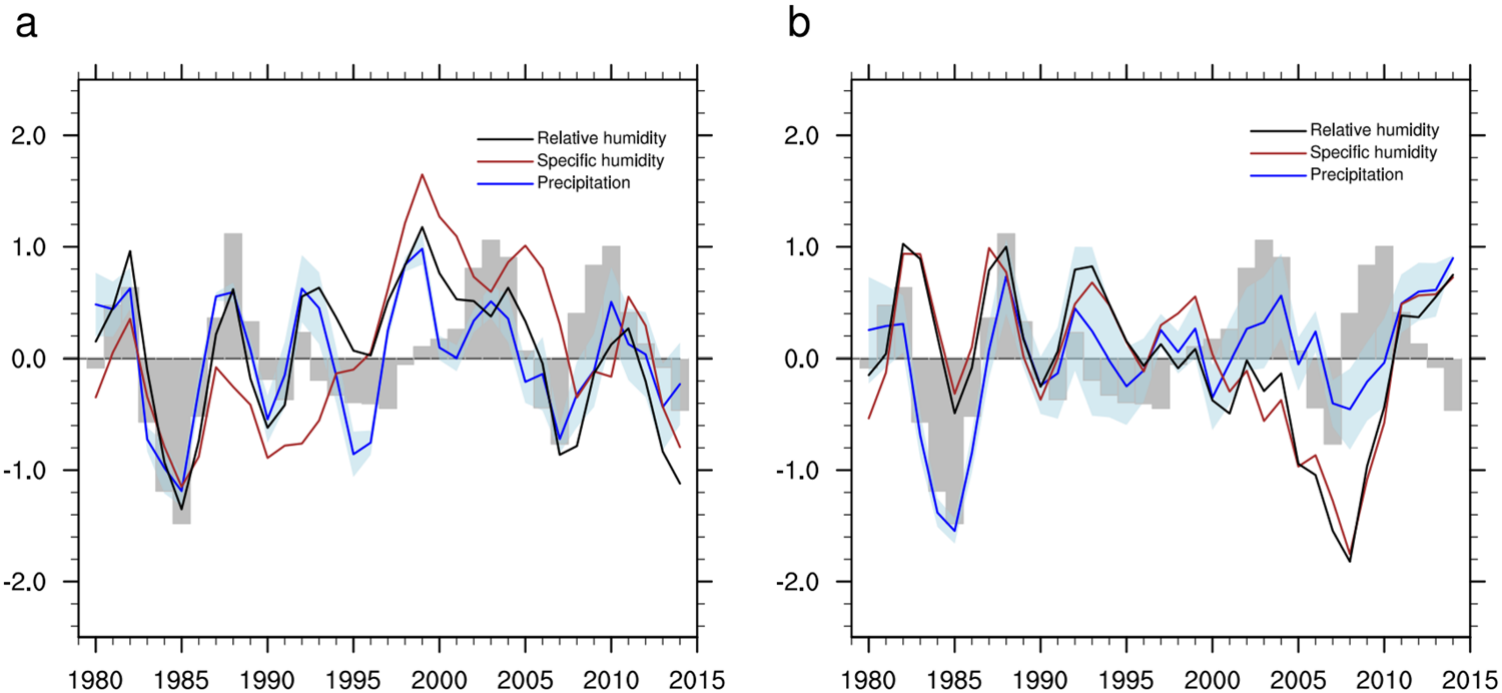
Time series of area-mean warm season precipitation, specific humidity, and relative humidity averaged over (**a**) the Tien Shan mountain ranges and (**b**) the basin areas. All data are normalized relative to the mean and standard deviation during the period 1979–2015. Three-point running means are applied to all time series.

In addition to large-scale dynamical and/or thermodynamical influences, precipitation may vary due to changes in the local environment^21,22^. One of the most prominent changes over ACEA in the past several decades is a sharp increase in near-surface temperature^8,17^. The change-point detection method identifies a significant shift in the mean state of lower tropospheric (500–925 hPa) temperature over AECA around 1997 (*p* < 0.01), with particularly pronounced warming over the Tien Shan mountain ranges. This is consistent with meteorological station observations, which indicate that temperatures over the Tien Shan have risen at a rate 2~3 times faster than the global average^8,20^. The water vapor saturation threshold in the atmosphere increases with temperature. Assuming relative humidity and circulation statistics to be approximately fixed, rainfall may thus be expected to increase with warming as the amount of water vapor in the atmosphere rises^17,20^. This relationship generally holds within the tropics, where oceanic sources of water vapor are abundant. However, it fails to hold if increases in moisture content do not keep pace with increases in water vapor saturation capacity. Relative humidity may thus decrease over land, especially in dry regions like Tien Shan that are located far from the oceans. Indeed, relative humidity over Tien Shan decreased substantially after 1998, due in part to strong warming and in part to a reduction in atmospheric moisture content (Figs 7 and S5). Warm season rainfall and lower tropospheric relative humidity over Tien Shan were highly correlated during 1979–2015 regardless of the choice of precipitation dataset (Supplementary Table S4). The reduction in warm season rainfall after 1998 over Tien Shan can therefore be interpreted as a response to changes in moisture content and temperature, which both act to reduce relative humidity. These changes contrast with those over basin areas, where cooling and moistening favor increased precipitation (Supplementary Fig. S5). The elevation-dependent warming within AECA can be attributed to the effects of albedo changes and associated surface-based feedbacks, changes in radiative flux, and aerosol effects^23–25^. The sources of the altitude-dependent changes in moisture content over AECA are less apparent, and require further study.

### Human context

Anthropogenic effects on the water cycle in AECA have intensified in recent decades^6^, including an extensive water diversion project initiated in 2000 that has transferred a large volume of water (23.27 × 10^8^ m^3^) from Bosten Lake (the largest inland freshwater lake in China) toward the Tarim basin^26,27^. The effects of this and other engineering projects on local water availability are reflected in increases in surface soil moisture and NDVI over the past 15 years (Fig. 3b,c). Along with cooler temperatures and increased moisture content at low levels (Supplementary Fig. S5), these changes in soil moisture^11^ and aboveground green biomass^13,27^ are hallmarks of enhanced moisture recycling within the basin areas. We hypothesize that sustained relatively high levels of warm season precipitation in the basin areas of AECA result from enhanced local moisture recycling, in part due to expanded irrigation^11,28^. This can be gleaned from the increasing evaporation rates observed by stations located in the basin areas (Supplementary Fig. 6). However, Bosten Lake and other sources of irrigation water fundamentally depend on meltwater from glaciers and snow in the Tien Shan mountain ranges^29^, indicating that enhanced precipitation in the basin areas has been maintained at the expense of the Tien Shan cryosphere. Indeed, studies indicate that GRACE-derived terrestrial water storage decreased dramatically over the Tien Shan mountain ranges^17^ while increasing over basin areas^30^ during the past decade.

**Figure 8:**
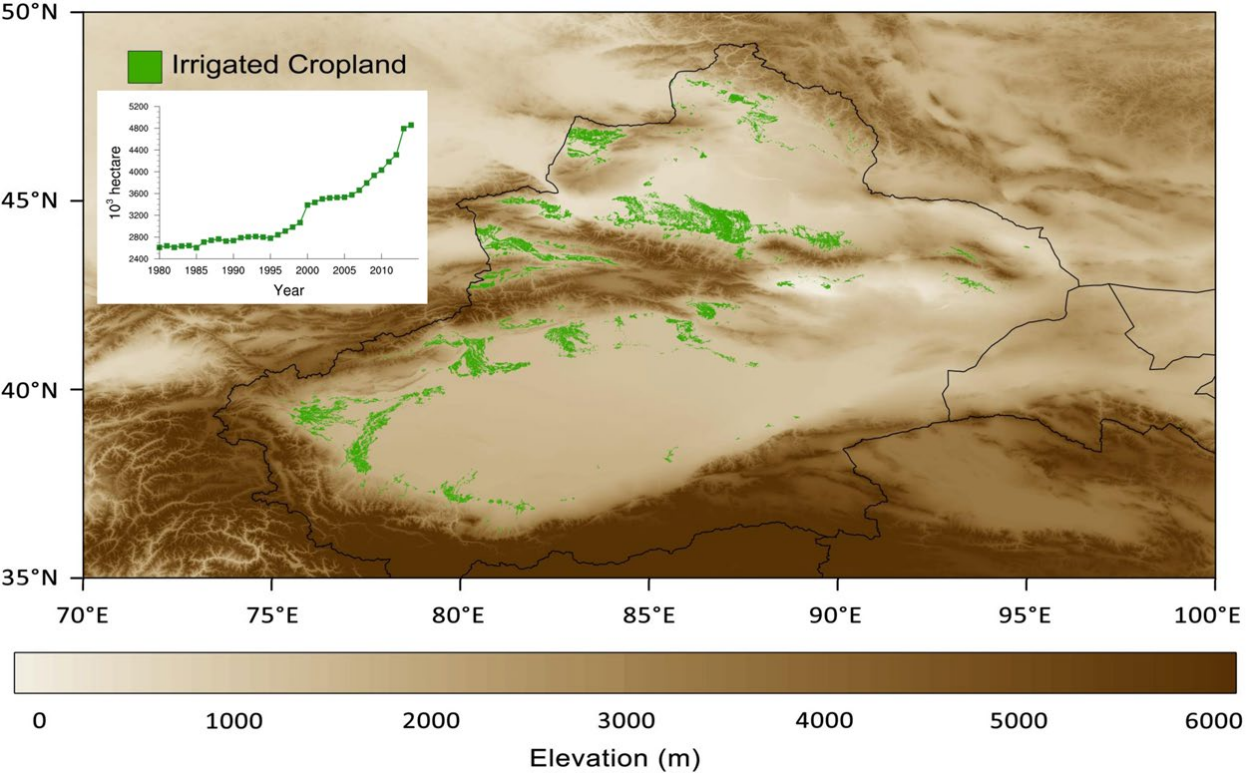
Spatial distribution of irrigated uplands (green areas) in Xinjiang province, China. The inset plot shows annual changes in irrigated area in Xinjiang province from 1980 through 2015.

Glaciers in the Tien Shan are expected to continue losing mass as climate warms even if precipitation increases^1^. Although glacier mass loss initially boosts available water resources via increased glacial runoff, river discharge will ultimately drop unless increases in other water sources (e.g., precipitation) compensate for the loss of the cryospheric buffer. Our results suggest that catchments with glaciated headwaters in the Tien Shan mountains, where water storage levels are expected to remain in deficit for the next half-century^17^, have already started to lose their cryospheric buffers. The promise of a new, wetter climate regime in AECA^13^ may thus be a mirage. Unless the loss of summertime precipitation within the Tien Shan is offset by increases in accumulation during the winter half year, glacier mass and snow cover will dwindle and potentially even disappear. Increases in precipitation over the basin areas supported by enhanced glacier and snow melt would reverse as those sources dry up, potentially resulting in a rapid drop in water resources within the basin areas with catastrophic implications for local agriculture and ecology. Anthropogenic activities like irrigation and groundwater exploitation, which re-allocate available water resources within the spatial domain, could exacerbate the impacts of this change. For example, the area of irrigated croplands in the Chinese portion of AECA has expanded rapidly since 1998, particularly along the periphery of the Tien Shan mountain ranges (Fig. 8). Current agricultural yields in this region rely heavily on irrigation, and would be unattainable with current technologies if irrigation levels cannot be maintained. Earlier studies indicate that this expansion of irrigation area has contributed to enhanced moisture recycling in this region^24,28^, but existing data are insufficient to support quantitative evaluations of the contribution of irrigation to precipitation outside of model simulations. In the meantime, future summer precipitation over AECA is projected to decrease by 4~7% by the middle of this century^1^. Although uncertainties remain, the available evidence consistently portends an increase in the frequency of dry summers. Resulting water shortages could intensify ecological problems and exacerbate political instability in AECA.

### Summary and outlook

Changes in the prevailing circulation and moisture transport patterns dominate variability in warm season precipitation over AECA, but local effects have grown increasingly influential in recent years. This shift is qualitatively robust across AECA, but with different expressions in the mountain and basin portions of the region. Both expressions are consistent with enhanced warming in the Tien Shan mountains after 1998. First, accelerated warming in the mountains favors reduced warm season precipitation locally. In addition to the moisture transport decrease over the mountain region, increases in saturation thresholds as climate warms will likely exacerbate the effects of decreases in atmospheric moisture content, leading to a decrease of warm season rainfall over the mountain ranges. Second, accelerated warming in the mountains favors increased warm season precipitation in the basin areas, as enhanced supplies of ice and snow meltwater to the basin areas (in tandem with expanded irrigation) intensify local moisture recycling. In the short term, the latter permits sustained or even increased precipitation in the basin areas despite reductions in moisture flux convergence. The evidence for these divergent changes in warm season precipitation is clear and supported by multiple independent data sets, but questions remain regarding the mechanisms behind the decrease in atmospheric moisture at high altitudes and the role of irrigation in the basin areas. Additional observations are urgently required, particularly those that can distinguish the relative contributions of meltwater, local recycling, and large-scale transport to precipitation in AECA.

This urgency is further compounded by the rapidly expanding human influence on the region. The population in Xinjiang, the largest province in China by area, increased by 80% between 1949 and 2011, while cultivated farmland increased by 63.5%^7^. These changes have accelerated the regional hydrological cycle^9,10^, and are expected to continue transforming the regional climate in the foreseeable future^1,24^, especially against the backdrop of the “*Silk Road Economic Belt*” project. This project is expected to bring extensive economic benefits to Central Asia, but associated rapid growth poses a potential threat to the sustainable management of regional water resources and their transboundary allocation^3,31^. Conflicting economic interests and societal needs have previously thwarted similar efforts at cooperation among the nations of Central Asia^3^. The participating nations should cooperate with all affected stakeholders to establish an effective and balanced framework for managing regional water resources as soon as possible. The regional disparities in rainfall change revealed in this study present a potentially severe challenge to the success of such a framework, and should be taken into consideration. Further analysis to clarify the mechanisms and potential future evolution of these disparities will be needed to support an effective and dynamic framework for sustainable development in this region.

## Methods

### Data

Precipitation data since 1961 are collected from 147 stations in AECA with records archived by the National Oceanic and Atmospheric Administration National Centers for Environmental Information (ftp://ftp.ncdc.noaa.gov/pub/data/noaa). Six gridded precipitation data sets are also used (see links provided in the footnotes to Supplementary Table S3, for availability.). Several atmospheric variables, including water vapor content and wind fields, are taken from the European Centre for Medium-Range Weather Forecasts (ECMWF) Interim (ERA-Interim) reanalyses^32^ (http://apps.ecmwf.int/datasets/). The results are qualitatively unchanged when the analysis is conducted with the Japanese 55-year Reanalysis (JRA-55)^33^ in place of ERA-Interim. Moderate Resolution Imaging Spectroradiometer (MODIS) retrievals of Normalized Difference Vegetation Index (NDVI)^34^ are used to track variations in soil wetness^35,36^, supplemented by satellite-derived soil moisture data produced by the European Space Agency (ESA) Climate Change Initiative (CCI)^37–39^. Other variables used in the analysis include river runoff from nine catchments with glaciated headwaters (Supplementary Table S2) and irrigation area from statistical yearbooks compiled by the National Bureau of Statistics of China (http://www.stats.gov.cn/tjsj/ndsj). Glaciers drawn in Fig. 1 are based on the GLIMS Glacier Database, Version 1, provided by the National Snow and Ice Data Center^40^.

### Glacier melting rate

Glacier measurements from 20 stations over Tien Shan mountain have been collected based on numerous previous studies^17^ (Fig. 4a and Supplementary Table 3). Generally, the area for each glacier is measured at three different time nodes. The first measurement timing ranges from the middle of 1950s to the early of 1990s, while the second and third measurement timings are more concentrated, around the year 2000 and 2012, respectively (Supplementary Table 3). The linear changing rates of each glacier are calculated during these two periods assuming the first measurement equals 100% of glacier as the reference year.

### Statistics

Uncertainties in mean values are estimated as twice the standard error of the mean. Differences between mean values are evaluated using a two-tailed Student’s *t* test. Trends are calculated using the robust Theil–Sen estimator^41,42^, in which the linear trend represents the median slope between all paired values. The Theil–Sen estimator is designed to reduce the effects of outliers and end points in linear trend analyses. Confidence intervals in the median slopes are calculated as proposed by Sen^42^. The quantitative and qualitative impacts of window size and start and end years are summarized in Fig. 2. The gradual increasing trend in warm season precipitation over the Tien Shan mountains after 1961 and its transition to a sharp decreasing trend in the late 1990s are robust for windows longer than 10 years. The transition is most pronounced for 1998, but remains significant for years between 1996 and 2003. Change points in climatological time series are evaluated using the sequential algorithm proposed by Rodionov^18^ with the cut-off length *l* set to 10 years. This algorithm is applied to both 10-year running means and 10-year running trends, where the latter are calculated using the Theil–Sen estimator. Confidence intervals in the mean value are approximately twice the standard error of the mean, with the factor adjusted according to the degrees of freedom. Spiegelhalter’s test has been used to confirm the suitability of the mean and standard error for describing climate shifts in warm season precipitation in AECA and its subregions. Only climate shifts that are significant at the 95% or greater confidence level are reported.

### Moisture Transport Calculation

Moisture transport is calculated on four different levels (850 hPa, 700 hPa, 600 hPa, and 500 hPa) to account for the complex topography of the AECA region (Supplementary Fig. S3). The calculated fluxes are used to examine how different moisture sources and circulation patterns contribute to variations in summer rainfall over AECA. Vertically integrated water vapor fluxes are calculated using horizontal wind (**u** = {*u*, *v*}) and specific humidity (*q*) via the equation

1 $${\bf{Q}}=\frac{1}{g}{\int }_{PT}^{PS}q{\bf{u}}dp,$$

where *g* is gravitational acceleration, *PS* is the surface pressure, *PT* is an upper bound (here 1 hPa). Horizontal winds and specific humidity can be expressed as ${\bf{u}}=\bar{{\bf{u}}}+{\bf{u}}^{\prime}$ and $q=\bar{q}+q^{\prime}$, where overbar and prime symbols denote the climatological means and the deviations from these climatological means during the analysis periods, respectively. Eq. (1) can be decomposed into four terms. Considering that $\frac{1}{g}{\int }_{PT}^{PS}\bar{q}\bar{{\bf{u}}}dp$ is stationary within the study periods while $\frac{1}{g}{\int }_{PT}^{PS}q{\bf{u}}dp$ is small in comparison to the terms involving climatological means, these two terms can be neglected^43^. Consequently, contributions to variations in the total moisture flux **Q** can be explored by calculating a moisture content (thermodynamic) component with varying specific humidity and time mean winds:.

2 $${{\bf{Q}}}^{\theta }=\frac{1}{{g}}{\int }_{{PT}}^{{PS}}{q}\bar{{\bf{u}}}{dp},$$

along with a large-scale circulation (dynamic) component with varying winds and time mean specific humidity:

3 $${{\bf{Q}}}^{\omega }=\frac{1}{g}{\int }_{PT}^{PS}\bar{q}{\bf{u}}dp,$$

Time mean wind and specific humidity are calculated from ERA-Interim reanalysis products for 1979–2014. Vertical integrals in Eqs. (1) through (3) are estimated from pressure level data using a trapezoidal rule. Horizontal water vapor flux divergence (∇∙**Q**) and its dynamic and thermodynamic components (∇∙**Q**^θ^ and ∇∙**Q**^ω^) are computed using central finite difference approximations via spherical harmonics.

## Electronic supplementary material

Supplementary Information

## Data Availability

Station records of precipitation are available from the National Oceanic and Atmospheric Administration National Centers for Environmental Information (ftp://ftp.ncdc.noaa.gov/pub/data/noaa). Gridded precipitation datasets are available through the links provided in the footnotes to Supplementary Table S4. The reanalysis products are available from the European Centre for Medium-Range Weather Forecasts (http://apps.ecmwf.int/datasets/). River runoff data can be provided upon request (please contact R.C. at crs2008@lzb.ac.cn for rivers 1–6 and Y.C. at chenyn@ms.xjb.ac.cn for rivers 7–9). Glacier area measurement data can be provided upon request (please contact Y.C. at chenyn@ms.xjb.ac.cn).

## References

[1] Sorg A, Bolch T, Stoffel M, Solomina O, Beniston M (2012). Climate change impacts on glaciers and runoff in Tien Shan (Central Asia). Nature Clim. Change.

[2] Farinotti D (2015). Substantial glacier mass loss in the Tien Shan over the past 50 years. Nature Geosci..

[3] Howard KWF, Howard KK (2016). The new “Silk Road Economic Belt” as a threat to the sustainable management of Central Asia’s transboundary water resources. Environ Earth Sci..

[4] Chatalova L, Djanibekov N, Gagalyuk T, Valentinov V (2017). The paradox of water management projects in Central Asia: An institutionalist perspective. Water.

[5] Pritchard HD (2017). Asia’s glaciers are a regionally important buffer against drought. Nature.

[6] Wan L (2015). Decadal climate variability and vulnerability of water resources in arid regions of Northwest China. Environment and Earth Science.

[7] Ling H, Xu H, Fu J, Zhang Q, Xu X (2012). Analysis of temporal-spatial variation characteristics of extreme air temperature in Xinjiang, China. Quaternary International.

[8] Li Z, Chen YN, Li WH, Deng HJ, Fang GH (2015). Potential impacts of climate change on vegetation dynamics in Central Asia. J. Geophys. Res..

[9] Shi Y (2007). Recent and future climate change in Northwest China. Climatic Change.

[10] Tao H, Gemmer M, Bai Y, Su B, Mao W (2011). Trends of streamflow in the Tarim River Basin during the past 50 years: Human impact or climate change?. J. Hydrol..

[11] Tao H, Borth H, Fraedrich K, Schneidereit A, Zhu X (2016). Hydrological extremes in the Aksu-Tarim River Basin: Climatology and regime shift. Clim. Dyn..

[12] Hua L, Zhong L, Ma Z (2017). Decadal transition of moisture sources and transport in northwestern China during summer from 1982 to 2010. J. Geophys. Res. atmos..

[13] Hong B (2014). Increasing summer rainfall in arid eastern-Central Asia over the past 8500 years. Sci. Rep..

[14] Aizen EM, Aizen VB, Melack JM, Nakamura T, Ohta T (2001). Precipitation and atmospheric circulation patterns at mid-latitudes of Asia. Int. J. Climatol..

[15] Aizen VB, Aizen EM, Melack JM, Dozier J (1997). Climatic and hydrologic changes in the Tien Shan, Central Asia. J. Clim..

[16] Zhao Y (2014). Impact of the middle and upper tropospheric cooling over central Asia on the summer rainfall in the Tarim Basin, China. J. Clim..

[17] Chen Y (2016). Changes in Central Asia’s Water Tower: Past, Present and Future. Sci. Rep..

[18] Rodionov SN (2006). Use of prewhitening in climate regime shift detection. Geophys. Res. Lett..

[19] Yasunari TJ, Koster RD, Lau WKM, Kim K-M (2015). Impact of snow darkening via dust, black carbon, and organic carbon on boreal spring climate in the Earth system. J. Geophys. Res. Atmos..

[20] Jiang Y (2013). Analysis on changes of basic climatic elements and extreme events in Xinjiang, China during 1961–2010. Adv. Clim. Change Res..

[21] Held IM, Soden BJ (2006). Robust responses of the hydrological cycle to global warming. J. Climate.

[22] Byrne MP, O’Gorman PA (2013). Link between land–ocean warming contrast and surface relative humidities in simulations with coupled climate models. Geophys. Res. Lett..

[23] Ghatak D, Sinsky E, Miller J (2014). Role of snow-albedo feedback in higher elevation warming over the Himalayas, Tibetan Plateau and Central Asia. Environ. Res. Lett..

[24] Pepin N (2015). Elevation-dependent warming in mountain regions of the world. Nature Clim. Change.

[25] Kraaijenbrink PDA, Bierkens MFP, Lutz AF, Immerzeel WW (2017). Impact of a global temperature rise of 1.5 degrees Celsius on Asia’s glaciers. Nature.

[26] Xu H, Ye M, Li K (2007). Changes in groundwater levels and the response of natural vegetation of transfer of water to the lower reaches of the Tarim River. Journal of Environmental Sciences..

[27] Sun Z, Opp C, Wang R (2009). Vegetation response to ecological water diversion in the lower Tarim river, Xinjiang, China. Basic Appl. Dryland Res..

[28] Wei J, Dirmeyer PA, Wisser D, Bosilovich MG, Bocko DM (2013). Where does the irrigation water go? An estimate of the contribution of irrigation to precipitation using MERRA. J. Hydrometeor..

[29] Zhou H, Chen Y, Perry L, Li W (2015). Implications of climate change for water management of an arid inland lake in Northwest China. Lake Reserv Manage..

[30] Yang P, Xia J, Zhan C, Qiao Q, Wang Y (2017). Monitoring the spatial-temporal changes of terrestrial water storage using GRACE data in the Tarim River basin between 2002 and 2015. Sci. Total Environ..

[31] Li P, Qian H, Horward K, Wu J (2015). Building a new and sustainable “Silk Road economic belt”. Environ. Earth Sci..

[32] Dee DP (2011). The ERA-Interim reanalysis: configuration and performance of the data assimilation system. Quart. J. Roy. Meteorol. Soc..

[33] Kobayashi S (2015). The JRA-55 Reanalysis: General specifications and basic characteristics. J. Meteor. Soc. Japan.

[34] Huete A (2002). Overview of the radiometric and biophysical performance of the MODIS vegetation indices. Remote Sens. Environ..

[35] Wang X, Xie H, Guan H, Zhou X (2007). Different responses of MODIS-derived NDVI to root-zone soil moisture in semi-arid and humid regions. J. Hydrol..

[36] Schnur MT, Xie H, Wang X (2010). Estimating root zone soil moisture at distant sites using MODIS NDVI and EVI in a semi-arid region of southwestern USA. Ecol. Inform..

[37] Liu YY (2011). Developing an improved soil moisture dataset by blending passive and active microwave satellite-based retrievals. Hydrol. Earth Sys. Sci..

[38] Liu YY (2012). Trend-preserving blending of passive and active microwave soil moisture retrievals. Remote Sens. Environ..

[39] Wagner, W. *et al*. Fusion of active and passive microwave observations to create an Essential Climate Variable data record on soil moisture, in: ISPRS Annals of the Photogrammetry, Remote Sensing and Spatial Information Sciences, vol. I-7, XXII ISPRS Congress, Melbourne, Australia (2012).

[40] Raup BH (2007). The GLIMS Geospatial Glacier Database: a New Tool for Studying Glacier Change. Global and Planetary Change.

[41] Theil H (1950). A rank-invariant method of linear and polynomial regression analysis I. Nederl. Akad. Wetensch., Proc..

[42] Sen P (1968). Estimates of the Regression Coefficient Based on Kendall’s Tau. Journal of the American Statistical Association.

[43] Wang Z, Duan A, Yang S, Ullah K (2017). Atmospheric moisture budget and its regulation on the variability of summer precipitation over the Tibetan Plateau. J. Geophys. Res. Atmos..

