# Publisher Correction: Regional disparities in warm season rainfall changes over arid eastern–central Asia

**DOI:** 10.1038/s41598-018-35361-z

**Published:** 2018-11-22

**Authors:** Wenhao Dong, Yanluan Lin, Jonathon S. Wright, Yuanyu Xie, Yi Ming, Han Zhang, Rensheng Chen, Yaning Chen, Fanghua Xu, Namei Lin, Chaoqing Yu, Bin Zhang, Shuang Jin, Kun Yang, Zhongqin Li, Jianping Guo, Lei Wang, Guanghui Lin

**Affiliations:** 1 Ministry of Education Key Laboratory for Earth System Modeling, Department of Earth System Science, and Joint Center for Global Change Studies (JCGCS), Tsinghua University, Beijing, 100084 China; 2 Geophysical Fluid Dynamics Laboratory, Princeton/NOAA, Princeton, New Jersey 08540-6649 USA; 3 Qilian Alpine Ecology and Hydrology Research Station, Key Laboratory of Inland River Ecohydrology, Northwest Institute of Eco-Environment and Resources, Chinese Academy of Sciences, Lanzhou, 730000 China; 4 State Key Laboratory of Desert and Oasis Ecology, Xinjiang Institute of Ecology and Geography, Chinese Academy of Sciences, Urumqi, 830011 China; 5 State Key Laboratory of Cryospheric Sciences/Tian Shan Glaciological Station, Cold and Arid Regions Environmental and Engineering Research Institute, Chinese Academy of Sciences, Lanzhou, 730000 China; 6 State Key Laboratory of Severe Weather and Key Laboratory of Atmospheric Chemistry of CMA, Chinese Academy of Meteorological Sciences, Beijing, 100081 China; 7 Key Laboratory of Tibetan Environment Changes and Land Surface Processes, Institute of Tibetan Plateau Research, Chinese Academy of Sciences (CAS), and the CAS Center for Excellence in Tibetan Plateau Earth Sciences, Beijing, 100101 China

Correction to: *Scientific Reports* 10.1038/s41598-018-31246-3, published online 29 August 2018

The original PDF version of this Article contained a truncated Figure 4. This error has now been corrected in the PDF version of the Article; the HTML version was correct from the time of publication.

