# Supplementary material for: Regional disparities in warm season rainfall changes over arid eastern–central Asia
Source: Sci Rep. 2018 Aug 29;8:13051. doi: 10.1038/s41598-018-31246-3 (PMC6115426; doi:10.1038/s41598-018-31246-3)
Supplement: Supplementary file 1 — Supplementary Information [file 41598_2018_31246_MOESM1_ESM.pdf]

Title: Regional disparities in warm season rainfall changes over arid eastern–central Asia

Authors: Wenhao Dong[1], Yanluan Lin[1★], Jonathon S. Wright[1★], Yuanyu Xie[1], Yi Ming[2], Han Zhang[1], Rensheng Chen[3], Yaning Chen[4★], Fanghua Xu[1], Namei Lin[1], Chaoqing Yu[1], Bin Zhang[1], Shuang Jin[5], Kun Yang[1], Zhongqin Li[5], Jianping Guo[6], Lei Wang[7], Guanghui Lin[1]

Legends:

Supplementary Figure S1. Distributions of surface topography (shading) and the (**a**) mean and (**b**) standard deviation of warm season rainfall (green contours) over AECA (black dash box) and surrounding areas. The mean and standard deviation of warm season rainfall are calculated for the period 1961–2000 based the APHRODITE dataset.

Supplementary Figure S2. Distributions of linear trends in warm season rainfall based on (**a**) APHRODITE during 1998–2007, (**b**) CMAP during 1998–2015, (**c**) GPCP during 1998–2015, (**d**) Delaware during 1998–2014, and (**e**) PERSIANN during 1998–2014. Stippling indicates that trends are significant at the 95% confidence level. All trends are calculated using the robust Theil–Sen estimator (see Methods). The dashed cyan contour bounds the Tien Shan mountain ranges and the dashed brown contour bounds the basin areas.

Supplementary Figure S3. a–**d**. Climatology of summertime moisture transport (shading) and wind fields (vectors) at (**a**) 850 hPa, (**b**) 700 hPa, (**c**) 600 hPa, and (**d**) 500 hPa averaged over 1979–2015 using ERA-Interim. Color scales and reference wind vectors vary by level. The black polygon indicates the AECA domain (Fig. 1).

Supplementary Figure S4. Distributions of moisture transport (shading) and wind fields (vectors) averaged for 1961–1987 (the first column), the difference between 1988–1998 and 1961–1987 (the second column), and the difference between 1999–2014 and 1988–1998 (the third column) on the 850 hPa (top row), 700 hPa (middle row), and 600 hPa (bottom row) isobaric surfaces. Pressure levels are masked where they descend below the land surface. Note that the first and second columns are based on ERA-40 reanalysis products, while the third column is based on ERA-Interim. Black dots/bold arrows in the second and third columns indicate trends of moisture content/wind fields that are statistically significant at the 95% confidence level.

Supplementary Figure S5. Vertical distributions of linear trends in ERA-Interim summer-mean (**a**) air temperature, (**b**) specific humidity, and (**c**) relative humidity averaged between 70 °E and 80 °E during 1998–2015. Horizontal distributions of linear trends in ERA-Interim summer-mean (**d**) air temperature, (**e**) specific humidity, and (**f**) relative humidity averaged between 925 hPa and 500 hPa during 1998–2015. Pressure levels are masked where they descend below the land surface. Trends are

calculated using the robust Theil–Sen estimator (see methods). Stippling indicates trends that are significant at the 95% confidence level. The boundary of the Tien Shan mountain ranges is marked as a cyan dashed line while the boundary of the basin areas is marked as a brown dashed line in panels **d** through **f**.

Supplementary Figure S6. Distributions of linear trends (circles) of evaporation from 38 stations located in basin areas in warm season overlaid with the linear trend of warm season precipitation during the period of 1998-2015. Trends in all stations are not significant at the 95% confidence level. The boundary of the Tien Shan mountain ranges is marked as a cyan solid line. The boundary of the basin areas is marked as a brown solid line.

Supplementary Table S1. Statistics of warm season rainfall variations based on different precipitation datasets, including correlations with station-based observations, mean±s.e.m., and linear trends during key time periods discussed in the text.

Supplementary Table S2. Detailed information for nine hydrological monitoring stations in AECA, including catchment area, geographic location, mean runoff during summer, and linear trends using the robust Theil–Sen estimator before and after 1998.

Supplementary Table S3. Detailed information for twenty glacier area measurement (units: km^2) in Tien Shan mountain ranges. Each glacier is measured at three different times, based on which the melting rates are calculated over the corresponding two different time periods.

Supplementary Table S4. Correlations between the SWJI and warm season rainfall over AECA based on six different precipitation analyses during 1979–2015.

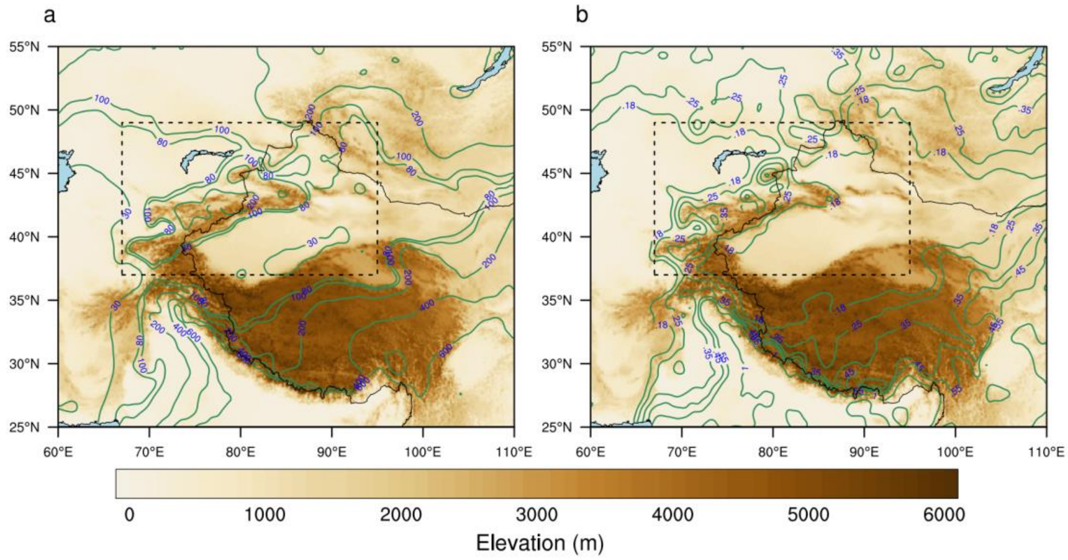

Supplementary Figure S1. Distributions of surface topography (shading) and the (**a**) mean and (**b**) standard deviation of warm season rainfall (green contours) over AECA (black dash box) and surrounding areas. The mean and standard deviation of warm season rainfall are calculated for the period 1961–2000 based the APHRODITE dataset.

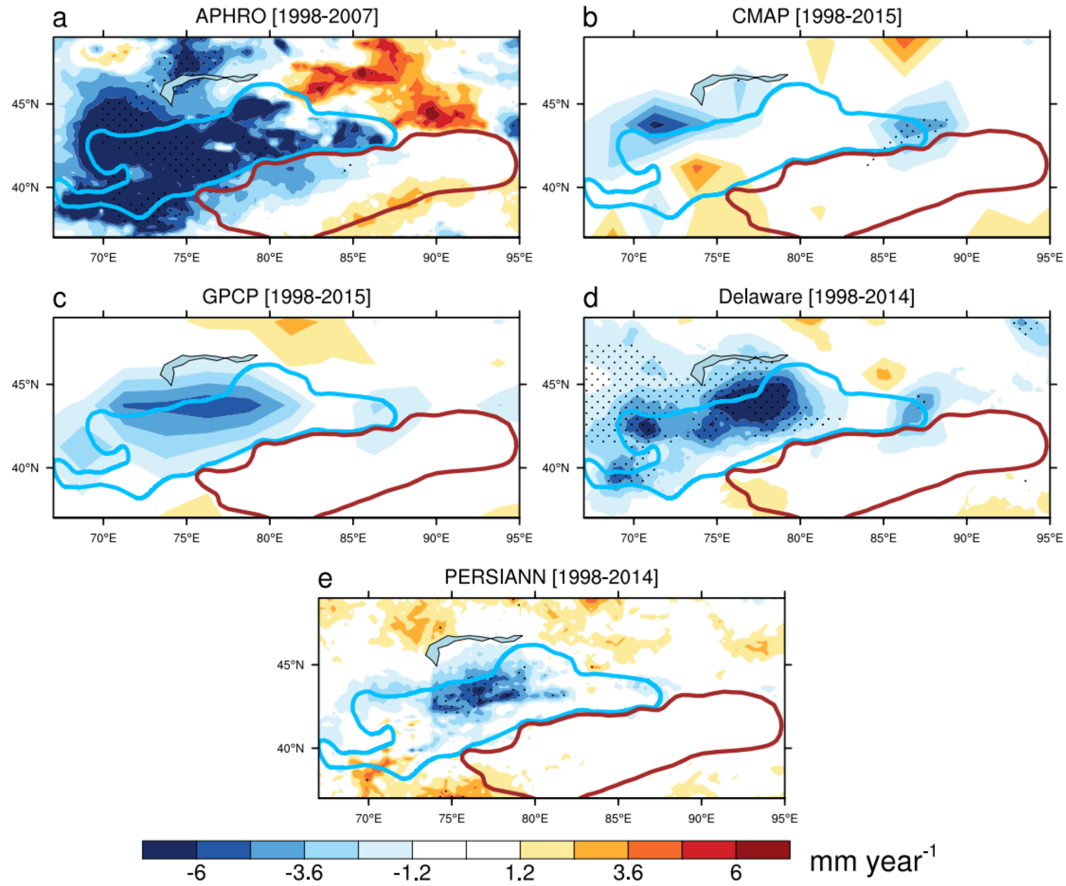

Supplementary Figure S2. Distributions of linear trends in warm season rainfall based on (**a**) APHRODITE during 1998–2007, (**b**) CMAP during 1998–2015, (**c**) GPCP during 1998–2015, (**d**) Delaware during 1998–2014, and (**e**) PERSIANN during 1998–2014. Stippling indicates that trends are significant at the 95% confidence level. All trends are calculated using the robust Theil–Sen estimator (see Methods). The dashed cyan contour bounds the Tien Shan mountain ranges and the dashed brown contour bounds the basin areas.

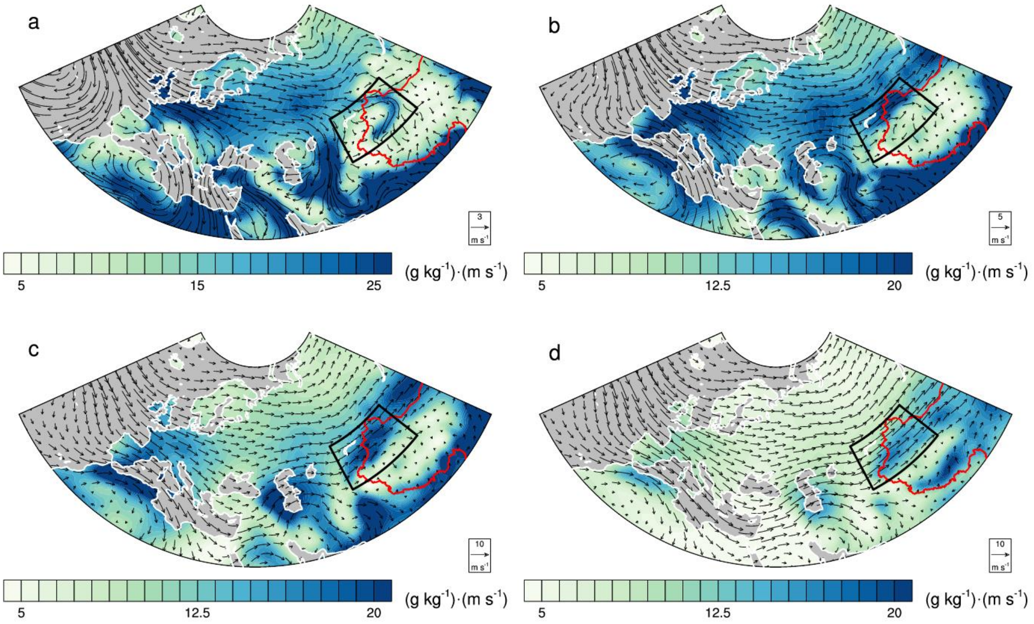

Supplementary Figure S3. a–**d**. Climatology of summertime moisture transport (shading) and wind fields (vectors) at (**a**) 850 hPa, (**b**) 700 hPa, (**c**) 600 hPa, and (**d**) 500 hPa averaged over 1979–2015 using ERA-Interim. Color scales and reference wind vectors vary by level. The black polygon indicates the AECA domain (Fig. 1).

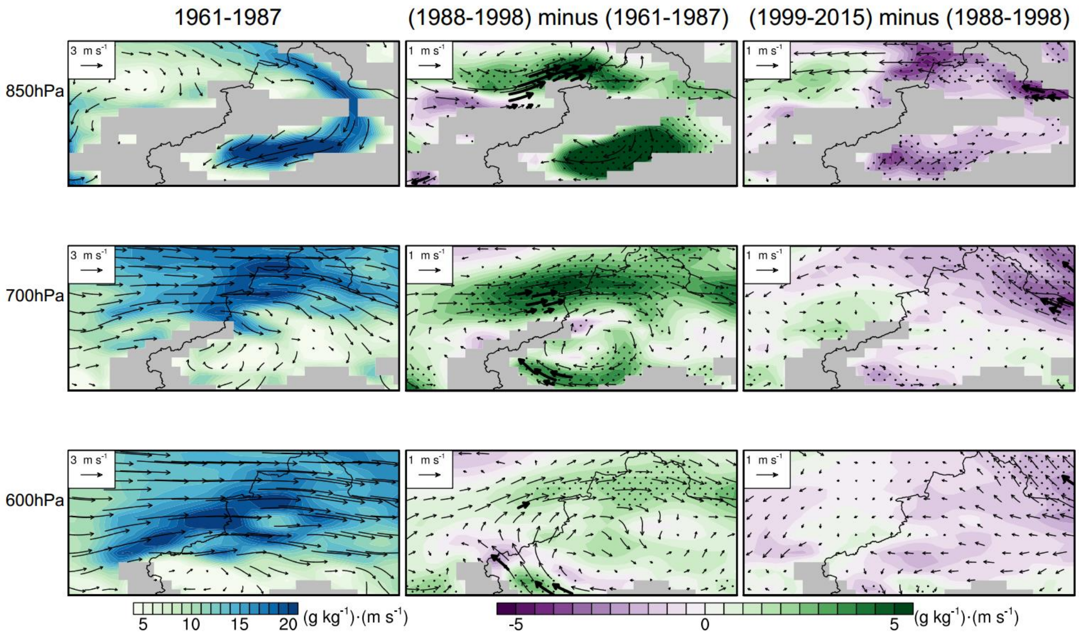

Supplementary Figure S4. Distributions of moisture transport (shading) and wind fields (vectors) averaged for 1961–1987 (the first column), the difference between 1988–1998 and 1961–1987 (the second column), and the difference between 1999–2014 and 1988–1998 (the third column) on the 850 hPa (top row), 700 hPa (middle row), and 600 hPa (bottom row) isobaric surfaces. Pressure levels are masked where they descend below the land surface. Note that the first and second columns are based on ERA-40 reanalysis products, while the third column is based on ERA-Interim. Black dots/bold arrows in the second and third columns indicate trends of moisture content/wind fields that are statistically significant at the 95% confidence level.

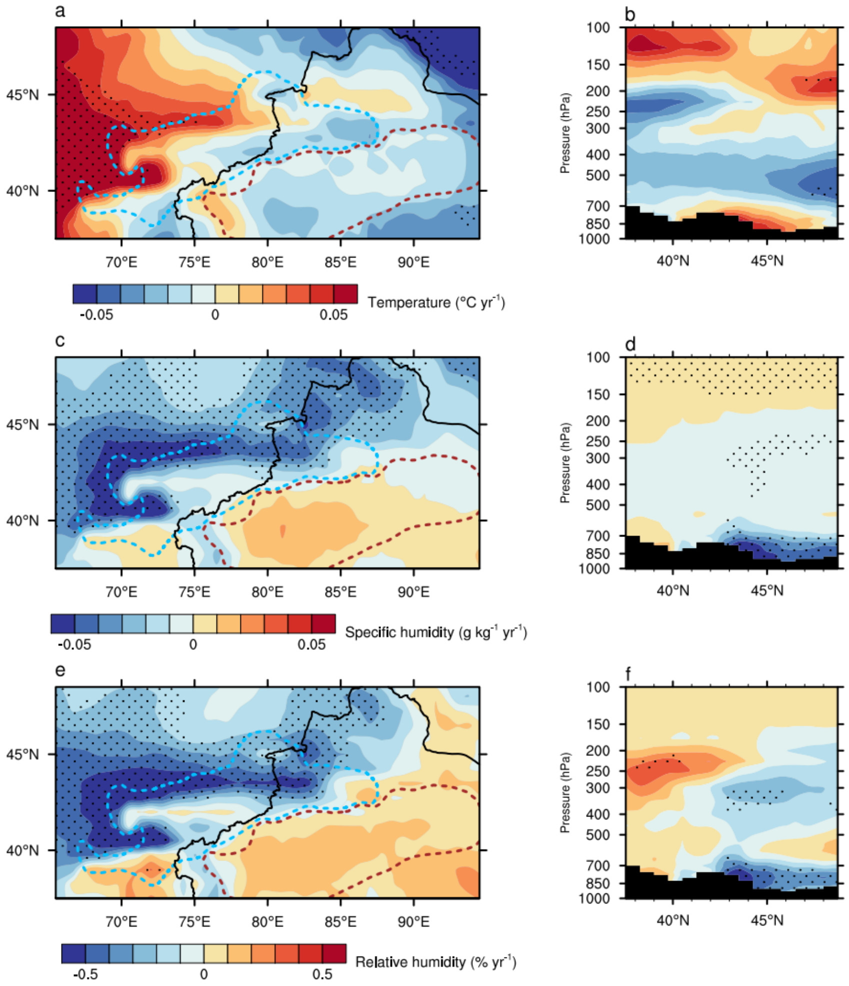

Supplementary Figure S5. Vertical distributions of linear trends in ERA-Interim summer-mean (**a**) air temperature, (**b**) specific humidity, and (**c**) relative humidity averaged between 70 °E and 80 °E during 1998–2015. Horizontal distributions of linear trends in ERA-Interim summer-mean (**d**) air temperature, (**e**) specific humidity, and (**f**) relative humidity averaged between 925 hPa and 500 hPa during 1998–2015. Pressure levels are masked where they descend below the land surface. Trends are calculated using the robust Theil–Sen estimator (see methods). Stippling indicates trends that are significant at the 95% confidence level. The boundary of the Tien Shan mountain ranges is marked as a cyan dashed line while the boundary of the basin areas is marked as a brown dashed line in panels **d** through **f**.

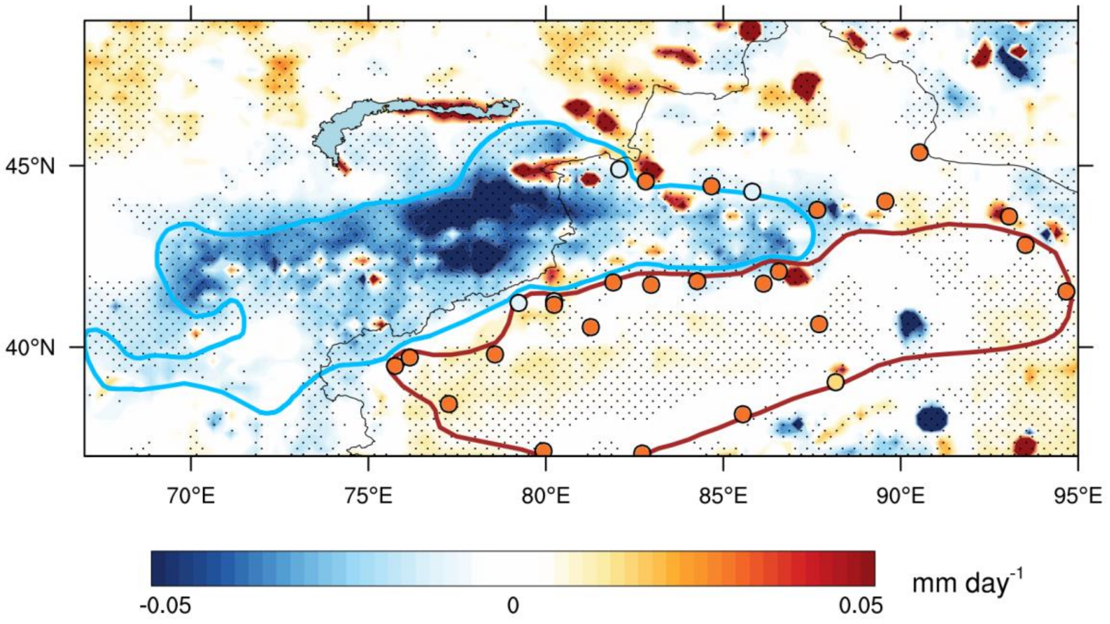

Supplementary Figure S6. Distributions of linear trends (circles) of evaporation from 38 stations located in basin areas in warm season overlaid with the linear trend of warm season precipitation during the period of 1998-2015. Trends in all stations are not significant at the 95% confidence level. The boundary of the Tien Shan mountain ranges is marked as a cyan solid line. The boundary of the basin areas is marked as a brown solid line.

Supplementary Table S1. Statistics of warm season rainfall variations based on different precipitation datasets, including correlations with station-based observations, mean ± s.e.m., and linear trends during key time periods discussed in the text.

Region	Datasets (Time)	Correlation with *in situ* observations	Mean (mm)			Linear trend (mm per decade)		
			1961–1986	1987–1997	1998–2015	1979–2015	1979–1997	1998–2014
AECA	Station (1961-2015)	**1.00**	82.01 ± 2.39	97.84 ± 5.20	94.71 ± 3.72	1.61	2.70	−6.01
	APHRO (1961-2007)	**0.95**	78.55 ± 2.97	92.25 ± 5.92	-	-	4.90	-
	TMPA (1998-2015)	**0.96**	-	-	116.63 ± 5.75	-		−5.63
	CMAP (1979-2015)	**0.91**	-	94.99 ± 5.35	93.58 ± 4.05	−0.37	3.22	−13.81
	GPCP (1979-2015)	**0.95**	-	120.54 ± 6.18	123.14 ± 4.92	3.28	7.61	−20.21
	Delaware (1961–2014)	**0.89**	89.86 ± 3.34	110.67 ± 7.91	102.20 ± 4.27	−0.40	6.44	−9.92
	PERSIANN (1983–2014)	**0.87**	-	110.52 ± 6.31	123.45 ± 4.75	-	-	−1.52
Tien Shan	Station (1961-2015)	**1.00**	82.23 ± 2.71	90.78 ± 5.72	89.13 ± 4.82	0.28	0.31	−20.58**
	APHRO (1961-2007)	**0.92**	81.83 ± 3.53	88.00 ± 6.23	-	-	−2.31	-
	TMPA (1998-2015)	**0.89**	-	-	110.74 ± 7.15			−21.02**
	CMAP (1979-2015)	**0.80**	-	93.40 ± 5.94	94.50 ± 4.84	0.22	3.16	−18.23
	GPCP (1979-2015)	**0.87**	-	117.07 ± 7.35	121.49 ± 5.83	0.79	4.04	−28.68*
	Delaware (1961–2014)	**0.91**	93.06 ± 5.15	103.75 ± 8.57	98.32 ± 5.56	−2.47	−0.31	−20.04*
	PERSIANN (1983–2014)	**0.72**	-	98.44 ± 7.32	122.10 ± 5.40	-	-	−10.89
Basin areas	Station (1961-2015)	**1.00**	82.35 ± 4.41	105.46 ± 5.93	98.88 ± 5.09	−0.69	3.50	−4.80
	APHRO (1961-2007)	**0.93**	70.69 ± 4.45	94.77 ± 6.46	-	-	8.02	-
	TMPA (1998-2015)	**0.95**	-	-	124.51 ± 6.63	-	-	5.50
	CMAP (1979-2015)	**0.86**	-	92.64 ± 6.00	94.76 ± 4.21	0.96	0.93	−11.29
	GPCP (1979-2015)	**0.86**	-	117.52 ± 6.91	124.65 ± 5.25	7.52	10.72	−11.74
	Delaware (1961–2014)	**0.83**	88.45 ± 4.08	115.88 ± 7.25	106.11 ± 4.69	1.95	15.69	1.1
	PERSIANN (1983–2014)	**0.84**	-	111.63 ± 7.71	125.17 ± 5.30	-	-	6.09

Notes: numbers with one star and two stars indicate trends that are significant at 90% and 95% confidence levels, respectively. Correlations in bold are significant at the 99% confidence level or greater. Dashes (-) mean that data were not available for the entirety of the specified period. Correlations with in situ observations are calculated after interpolating gridded analyses to station locations during the respective periods of overlap. Linear trends are calculated using the robust Theil–Sen estimator (see Methods)

Supplementary Table S2. Detailed information for nine hydrological monitoring stations in AECA, including catchment area, geographic location, mean runoff during summer, and linear trends using the robust Theil–Sen estimator before and after 1998.

	Name Period	Area (km^2)	latitude (°N)	longitude (°E)	Height (m)	Runoff ($m^3 s^{-1}$)	Trend (% yr^{-1}) 1980–1998	Trend (% yr^{-1}) 1998–2011
1	Shaliguilanke [1961–2011]	19166	40.95	78.60	1909	179.48	1.86**	–4.87**
2	Xiehela [1961–2011]	12816	41.72	79.62	1427	4325.77	0.81	–3.33**
3	Kuche [1961–2011]	3118	41.90	83.07	1280	25.56	2.27*	–3.35*
4	Hutubi [1961–2012]	1840	43.79	86.59	1282	31.39	1.42*	–3.26*
5	Glacier #1 [1980–2014]	3.34	43.19	86.82	3843	8.53	2.22*	–3.26
6	Urumuqi [1961–2012]	924	43.37	87.20	1942	15.81	1.33**	–1.83
7	Xidaqiao [1961–2015]	18624	42.22	85.73	1340	382.75	1.06	–3.34**
8	Dashankou [1972–2008]	4424	42.45	86.23	1320	178.19	0.94*	–2.40*
9	Huangshuigou [1961–2011]	1057	43.92	86.05	900	17.64	2.52**	–6.12

Note: numbers with one star and two stars indicate trends that are significant at 90% and 95% confidence levels, respectively.

Trend for the *Dashankou* over 1998−2011 is calculated during the period of 1998−2008 instead.

Supplementary Table S3. Detailed information for twenty glacier area measurement (units: km^2) in Tien Shan mountain ranges. Each glacier is measured at three different times, based on which the melting rates are calculated over the corresponding two different time periods.

No	Name	Area (measured year)		
1	Tomur	421.27 (1976)	409.98 (2000)	404.84 (2011)
2	North	334.22 (1990)	319.49 (2000)	280.73 (2011)
3	West	2248.16 (1990)	2135.78 (2000)	1983.38 (2011)
4	West-central	55.11 (1990)	47.78 (2000)	39.83 (2011)
5	East-central	550.55 (1990)	501.83 (2000)	407.16 (2011)
6	Terisakkan	14.1(1956)	6.5 (2001)	5.1 (2012)
7	Koksu	108.6(1956)	64.1 (2001)	56.1 (2012)
8	Chizhin	8.7(1956)	4.2 (2001)	3.8 (2012)
9	Kora	67.8(1956)	47.5 (2001)	44.2 (2012)
10	Pskem	210.63(1970)	177 (2000)	168.15 (2007)
11	lli-Kungoy	632.8(1970)	565 (2000)	542.4 (2007)
12	At-Bashy	114.24(1970)	102 (2000)	97.92 (2007)
13	SE-Fergana	248.52(1970)	228 (2000)	228 (2007)
14	Lower Nargn	83 (mid-1970s)	77.19 (mid-1990s)	74.87 (mid-2000s)
15	Dzhetim	532 (mid-1970s)	409.64 (mid-1990s)	364.58 (mid-2000s)
16	Akshiirak	204 (mid-1970s)	179.52 (mid-1990s)	177.72 (mid-2000s)
17	At-Bashi Kirkasi	151 (mid-1970s)	129.86 (mid-1990s)	128.56 (mid-2000s)
18	Urumqi No.1	1.87 (1976)	1.742 (2000)	1.619 (2012)
19	TS.TUYUKSUYSKIY	3.17 (1965)	2.549 (2000)	2.297 (2012)
20	Abramov	24.64 (1968)	24.03 (2000)	23.91 (2014)

Supplementary Table S4. Correlations between the SWJI and warm season rainfall over AECA based on six different precipitation analyses during 1979–2015.

Regions	Variables	Periods	APHRODITE[1]	TMPA[2]	CMAP[3]	GPCP[4]	Delaware[5]	PERSIANN[6]
Tien Shan	Specific humidity	1979–2015	0.32	0.61**	0.53**	0.52**	0.29*	0.52**
		1979–1998	0.28	/	0.55**	0.37	0.36	0.32
		1998–2015	0.53*	0.61**	0.25	0.52**	0.76**	0.41*
	Relative humidity	1979–2015	0.71**	0.82**	0.72**	0.78**	0.80**	0.69**
		1979–1998	0.70**	/	0.82**	0.78**	0.80**	0.73**
		1998–2015	0.89**	0.82**	0.61**	0.81**	0.92**	0.72**
Basin areas	Specific humidity	1979–2015	0.17	0.71**	0.24	0.03	0.34**	0.10
		1979–1998	0.27	/	0.13	0.32	0.22	0.58**
		1998–2015	0.31	0.71**	0.63**	0.53**	0.67**	0.54**
	Relative humidity	1979–2015	0.31	0.78**	0.36**	0.00	0.46**	0.15
		1979–1998	0.49**	/	0.35	0.49**	0.45*	0.70**
		1998–2015	0.58*	0.78**	0.75**	0.74**	0.72**	0.71**

Notes: numbers with one star and two stars indicate statistical significance at the 90% and 95% confidence levels, respectively. Correlations are calculated for overlapping periods, and are only reported when these overlapping periods exceed 10 years.

[1] http://www.chikyu.ac.jp

[2] https://pmm.nasa.gov/data-access/downloads/trmm

[3] https://www.esrl.noaa.gov/psd/data/gridded/data.cmap.html

[4] https://www.esrl.noaa.gov/psd/data/gridded/data.gpcp.html

[5] https://www.esrl.noaa.gov/psd/data/gridded/data.UDel_AirT_Precip.html

[6] ftp://persiann.eng.uci.edu/CHRSdata/PERSIANN/mthly

References

1. Yatagai, A. *et al.* APHRODITE: Constructing a long-term daily gridded precipitation dataset for Asia based on a dense network of rain gauges, *Bull. Am. Meteorol. Soc.*, **93**, 1401–1415 (2012).
2. Huffman, G. J. *et al.* The TRMM Multisatellite Precipitation Analysis (TMPA): Quasi-global, multiyear, combined-sensor precipitation estimates at fine scales, *J. Hydrometeorol.*, **8**, 38–55 (2007).
3. Xie, P. & Arkin, P. A. Global precipitation: a 17-year monthly analysis based on gauge observations, satellite estimates, and numerical model outputs. *Bull. Am. Meteor. Soc.* **78**, 2539–2558 (1997).
4. Adler, R. F. *et al.* The version 2 Global Precipitation Climatology Project (GPCP) monthly precipitation analysis (1979-Present). *J. Hydrometeor.* **4**, 1147–1167 (2003).

5. Matsuura, K. & Willmott, C. J. Terrestrial precipitation: 1900–2014 gridded monthly time series. *Center for Climatic Research Department of Geography Center for Climatic Research, University of Delaware*. http://climate.geog.udel.edu/~climate/html_pages/Global2014/README.GlobalTsP2014.html (2015).
6. Ashouri, H. *et al.* PERSIANN-CDR: Daily Precipitation Climate Data Record from Multisatellite Observations for Hydrological and Climate Studies. *Bull. Am. Meteor. Soc.* **96**, 69–83 (2014).
